# The Role of Metabolomics in Current Concepts of Organ Preservation

**DOI:** 10.3390/ijms21186607

**Published:** 2020-09-10

**Authors:** Mindaugas Kvietkauskas, Viktorija Zitkute, Bettina Leber, Kestutis Strupas, Philipp Stiegler, Peter Schemmer

**Affiliations:** 1 General, Visceral and Transplant Surgery, Department of Surgery, Medical University of Graz, Auenbruggerpl. 2, Graz 8036, Austria; min.kvietkauskas@gmail.com (M.K.); viktorijazitkute@gmail.com (V.Z.); bettina.leber@medunigraz.at (B.L.); peter.schemmer@medunigraz.at (P.S.); 2 Faculty of Medicine, Vilnius University, M. K. Ciurlionio 21, 03101 Vilnius, Lithuania; kestutis.strupas@santa.lt

**Keywords:** transplantation, machine perfusion, metabolomics, preservation, static cold storage

## Abstract

In solid organ transplantation (Tx), both survival rates and quality of life have improved dramatically over the last few decades. Each year, the number of people on the wait list continues to increase, widening the gap between organ supply and demand. Therefore, the use of extended criteria donor grafts is growing, despite higher susceptibility to ischemia-reperfusion injury (IRI) and consecutive inferior Tx outcomes. Thus, tools to characterize organ quality prior to Tx are crucial components for Tx success. Innovative techniques of metabolic profiling revealed key pathways and mechanisms involved in IRI occurring during organ preservation. Although large-scale trials are needed, metabolomics appears to be a promising tool to characterize potential biomarkers, for the assessment of graft quality before Tx and evaluate graft-related outcomes. In this comprehensive review, we summarize the currently available literature on the use of metabolomics in solid organ Tx, with a special focus on metabolic profiling during graft preservation to assess organ quality prior to Tx.

## 1. Introduction

Solid organ transplantation (Tx) is the only curative treatment option for patients suffering from end-stage organ failure. Liver, kidney, heart, lung and, to some extent, pancreas and intestine Tx are incorporated into routine clinical care worldwide, and both patient and allograft survival are continuing to improve [1]. However, the growing disparity between organ supply and demand has led to the increasing use of donation after circulatory death (DCD) and extended criteria donor (ECD; aged ≥60 years or aged 50–59 years with vascular comorbidities) allografts [2,3,4], despite their higher susceptibility to ischemia-reperfusion injury (IRI) and consecutive inferior outcomes, including mortality and morbidity after Tx [5,6].

The organ preservation process is a critical link in the chain of donation and Tx, and therefore is of major interest in research in order to provide strategies to improve Tx outcome [7]. The allograft, metabolically impaired during warm and cold ischemia (WI and CI), is further damaged by a paradox reperfusion injury after revascularization and re-oxygenation. Short-term and long-term complications including post-reperfusion syndrome, delayed graft function (DGF) and even immune activation have been associated with IRI [5,8].

The implementation of new storage techniques, such as machine perfusion (MP), has paved the way for the continuous supply of oxygen and substrates for the synthesis of adenosine triphosphate (ATP) and other metabolites, enabling the continuous removal of end products and stimulation of the organ’s metabolism [9]. Studies have demonstrated reduced rates of DGF and improved allograft survival in machine-perfused organs compared to static cold storage (SCS) [4,10,11,12]. Moreover, MP provides a unique opportunity to collect graft tissue and perfusate samples, as well as information regarding functional activity and flow dynamics prior to Tx. The accurate evaluation of allograft quality is essential in order to prevent unjustified donor organ rejection [13,14], and to estimate Tx outcomes. Additionally, MP offers a unique platform to facilitate intervention and modification to further optimize ECD grafts [5,15]. However, it remains unclear which temperature setting is preferable for optimal organ preservation [16]. Hypothermia slows metabolism and oxygen consumption, so that organs can survive longer without nutrient supplements, while normothermia preserves the graft at a near-physiological condition [5].

Metabolomics, firstly introduced in the late 1990s, is a postgenomic high-throughput systems biology approach of diagnostic innovation in clinical medicine [17,18]. In metabolomics, a large number of metabolites (sugars, amino acids, lipids, organic acids and nucleotides) can be measured using non-chemical, non-colorimetric methods, such as gas chromatography-mass spectrometry (GC-MS), liquid chromatography–MS (LC-MS) or nuclear magnetic resonance (NMR) spectroscopy [18,19]. The advantages of these analytical approaches are their accuracy, rapidity, the small sample volume, and the possibility of simultaneous detection and quantification in a single measurement, mostly without any preselection [20]. NMR spectroscopy relies on the signals from various nuclei, including ^1^H, ^31^P and ^13^C, while MS involves the ionization of metabolites present in samples and separation based on the mass/charge ratio [21,22]. Moreover, MS can also be combined with chromatographic methods (LC or GS) to improve metabolite separation. These technologies allow the investigation of metabolic changes in disease models and organ physiology, including Tx [23].

In general, metabolomic approaches have been performed to monitor two key aspects of organ physiology during preservation: (i) severity of organ IRI and (ii) organ function or dysfunction [19]. Several metabolomics studies revealed altered levels of metabolites originating from the urea cycle (urea and glutamate), energy metabolism (e.g., formate, orthophosphate, ATP, lactate, pyruvate, fatty acids and carbohydrates), oxidative phosphorylation (fumarate and succinate) and oxidative stress (increased levels of reduced glutathione) in IRI [19,22].

With a better understanding of the underlying harmful metabolic processes occurring during organ preservation, the possibility of ameliorating organ quality, as well as extending storage time and even improving allograft quality prior to Tx, may become clinical routine. It seems that innovative techniques, such as MP, combined with metabolomics has significant potential as a clinical tool for the assessment of preserved organs before Tx, since many potential biomarkers could be identified with the evolution of metabolomics. However, the current level of evidence is scarce and further studies are needed to pave the way for clinical trials. The purpose of this comprehensive review is to give an overview of the literature on the use of metabolomics in solid organ Tx, with special focus on metabolic profiling during graft preservation in order to compare preservation methods and assess the quality of grafts prior to Tx.

## 2. Materials and Methods

This comprehensive literature review was performed by selecting articles investigating different solid organ preservation methods for Tx, in which analytical techniques of metabolomics were applied. A literature search was conducted in the MEDLINE and EMBASE databases using Medical Subject Heading (MeSH) terms “metabolomics”, “heart”, “lung”, “kidney”, “liver”, “intestine”, “pancreas”, and “transplantation” until and including 10 May 2020 (English articles only). All hits were screened by title and abstract by two reviewers (MK and VZ) independently. Then full-text articles were reviewed for potential eligibility. Additional articles identified through reference list screening were included. Database-specific search strategies and a flowchart of the literature search according to the PRISMA guideline are provided as Supplementary Materials [24]. In total, 38 publications were included in this study.

## 3. Comprehensive Review

### 3.1. Metabolomics in Heart Preservation

The shortage of donor hearts relative to the demand is an ongoing challenge, given the increasing societal burden of heart failure [25]. A large number of potential donor hearts are discarded because the short safe preservation time of 4–6 h is exceeded due to logistical reasons [26,27]. The standard SCS method is simple and cheap, but suboptimal for preserving cardiac allografts, especially in the case of ECD hearts [26]. Research is currently focusing on the development of new preservation strategies to enhance the preservation of donor hearts, extend the maximum preservation duration, and evaluate the quality of donor hearts prior to Tx [27]. MP provides a continuous perfusion with oxygenated blood or preservation solution, resulting in improved metabolism compared to SCS (Table 1).

Previous experiments by Peltz et al. suggested significant advantages in rat hearts preserved by hypothermic MP (HMP; 4–10 °C) over SCS [28] due to improved cellular ATP and energy charge levels during the ischemic period [28]. In a canine study, NMR spectroscopy analysis revealed a dramatic decrease in tissue lactate in hearts preserved with continuous HMP with similar levels of myocardial edema [29]. After SCS, a more than five-fold increased lactate to alanine ratio was observed when compared to preservation by HMP [29]. Comparable results have been described in large animal experiments performed on pig hearts [30]. Continuous perfusion reduces the functional impairment of myocardium and tissue lactate accumulation without increasing edema; it therefore appears to be a promising tool to improve the results of heart Tx.

In 2010, Cobert et al. compared oxidative metabolism during 10 h of canine heart HMP with two commonly used extracellular-type preservation solutions (UW and Celsior^®^) [31]. Despite increased edema development, no detriment to the metabolic profile, analyzed by ^1^H NMR spectroscopy of tissue samples, was observed in the Celsior^®^ group. Lactate/alanine ratios remained low in both groups, denoting favorable metabolic profiles in HMP and indicating primarily aerobic metabolism. Moreover, lactate accumulation in the preservation solution was low and did not increase over time in either group [31,32]. Elevated lactate/alanine ratios in the right ventricle and left atrium stood in contrast to the low ratios found in the left ventricle. This data suggested that, despite excellent left ventricular perfusion, right ventricle perfusion is reduced, and oxidative metabolism may not be maintained by retrograde HMP [32].

A previously published study investigated myocardial metabolism on isolated rat hearts during HMP [33]. The authors found that glucose, even if provided at high concentrations, is minimally effective on oxidative pathways during MP. Pyruvate appears to be a more promising exogenous substrate, as the significantly increased incorporation of labeled carbon into Krebs cycle intermediates, in positions that exclusively occur through oxidative metabolism, could be demonstrated via a ^1^H and ^13^C NMR spectroscopy approach [33]. Experiments with discarded human hearts showed that HMP could support myocardial metabolism over long periods [34]. The lactate/alanine ratios determined by ^1^H NMR spectroscopy were lower in all perfused hearts when compared to the SCS group, indicating ongoing oxidative metabolism and reduced intracellular lactate accumulation in the MP groups. Moreover, ^31^P NMR spectroscopy demonstrated more stable high-energy phosphate to inorganic phosphate ratios in the perfusion groups, indicating that HMP preservation is effective in maintaining myocardial high-energy phosphates even over 12 h of perfusion [34]. This study demonstrated that the acceptable ischemic interval of donor hearts could be increased using MP techniques; therefore, improved donor–recipient matching and extension of the donor pool could be achieved by permitting for long-distance procurements.

Recently, Martin et al. compared the metabolic changes during WI and CI in mice, as well as in porcine and human hearts using LC-MS [35]. They proposed that succinate accumulation is a major feature within ischemic hearts across species, and that CI slows succinate generation, thereby reducing tissue damage upon reperfusion caused by the production of mitochondrial reactive oxygen species (ROS). Importantly, the inevitable periods of WI during organ procurement lead to the accumulation of damaging levels of succinate, despite cooling organs as rapidly as possible [35]. Moreover, the metabolism during WI and CI was similar in hearts of different species, encouraging the development of therapies using animal models. The data suggest that preventing oxidation and the accumulation of succinate during Tx might improve the outcomes of Tx. This could pave the way towards new treatment approaches.

However, there is a lack of clinical data, and more trials are needed in order to evaluate the role of metabolomics and the possibility of ameliorating graft quality prior to heart Tx.

### 3.2. Metabolomics in Lung Preservation

The field of lung Tx has made significant advances over the last decades. Despite these advances, morbidity and mortality remain high when compared to other examples solid organ Tx [36]. Ex vivo lung perfusion is already well established in clinical routine, and allows explanted donor lungs to be perfused and ventilated while being evaluated and reconditioned prior to Tx [36,37]. Advanced knowledge about the metabolic profile during preservation can help in finding innovative biomarkers for early allograft dysfunction (EAD), enabling timely therapeutic intervention to prevent functional decline. Currently, there is only a limited number of available metabolomics studies in lung preservation for Tx (Table 2).

Pillai et al. showed, as early as 1986, the feasibility of obtaining ^31^P NMR spectra of porcine lungs maintained in a viable state during normothermic MP (NMP; 37 °C) with oxygenated blood [38]. During anoxia or ischemia, ATP and intracellular pH declined and inorganic phosphate increased, but all returned to control levels during subsequent normoxia or reperfusion [38]. Deep knowledge on the recovery of the lungs from anoxia and ischemia is important in order to improve the protocols for preservation.

In 2003, Jayle et al. presented the beneficial effects of polyethylene glycol (PEG) in lung cold preservation [39]. In their study, PEG preserved porcine lungs better than UW and Euro-Collins (EC) solution. By means of ^1^H NMR spectroscopy, lactate, pyruvate, citrate and acetate were only detected after reperfusion, with a reduced production of acetate and pyruvate in PEG-preserved organs indicating a better mitochondria metabolism and integrity [39]. As a result, PEG solution was able to improve the pulmonary vascular resistance and reduce leukocyte infiltration (the important factors related to IRI).

Peltz et al. characterized lung metabolism in rats by ^13^C NMR spectroscopy, and suggested that glucose added to lung preservation solutions plays a minor role as an energy source [40,41]. Instead, the lung prefers to catabolize endogenous fuels during the SCS period. Adding a substrate such as pyruvate leads to multiple metabolic alterations, including the following: (i) enhanced overall oxidative metabolism, (ii) reduction of the contribution of endogenous stores, and (iii) activation of glucose and glycogen synthesis [40]. To sum up, the addition of pyruvate to Perfadex^®^ solution increased metabolism during SCS and improved lung function after reperfusion [40,41]. The development of metabolomic techniques allowed the exploration of the preservation solution substrate’s composition’s influence on graft metabolism during storage. Further research could help to extend the ischemic interval of stored lungs and improve the results of lung Tx.

In 2012, Benahmed et al. assessed the tissue quality of DCD pigs’ lungs using ^1^H NMR spectroscopy [42]. They identified 35 mostly upregulated metabolites over the period of SCS, indicating cellular degradation, whereas levels of glutathione decreased. During HMP, the majority of the metabolites remained stable, including glutathione. In contrast, the levels of uracil showed a reverse profile, indicating cell damage followed by oxidative stress. These results demonstrated that HMP has a positive effect on lung quality by protecting cells against oxidative disorders. Moreover, glutathione and uracil were found to be promising biomarkers for the evaluation of lung quality prior to Tx [42]. The authors described NMR to be a very reliable and rapid technique, which can be simply implemented in a hospital environment.

More recently, Hsin et al. revealed a small panel of metabolites, such as N2-methylguanosine, 5-aminovalerate, oleamide and decanoylcarnitine, in the perfusate highly correlating with primary graft dysfunction (PGD). These metabolites were identified as potential biomarkers for the selection of human ECD lungs after 4 h of ex vivo lung NMP [43]. However, further validation studies are needed to confirm these findings. By identifying high risk lung grafts, it may be possible to develop ex vivo repair strategies, using the MP platform, to render these lungs suitable for Tx.

### 3.3. Metabolomics in Kidney Preservation

Kidney Tx indisputably confers a significant survival advantage and a better quality of life compared to dialysis [44]. Currently, there is increasing evidence supporting the use of pulsatile MP over SCS in kidney preservation [4], but more studies are needed to compare MP and SCS. Previous studies suggested that metabolomics might be a useful method of evaluating renal medullary damage ex vivo after CI and reperfusion from tissue, plasma, urine and perfusate samples, showing more efficient results than conventional histology and biochemical analysis (Table 3).

Early experimental studies on porcine kidneys compared two standard preservation solutions, UW and EC, for kidney SCS (24 and 48 h) [20,45,46]. The most relevant metabolites for evaluating kidney function after autoTx, determined by ^1^H NMR spectroscopy, were citrate, dimethylamine (DMA), lactate and acetate in urine, and trimethylamine-N-oxide (TMAO) in urine and plasma. While the TMAO/creatinine, DMA/creatinine, lactate/creatinine and acetate/creatinine ratios were significantly higher in kidneys stored in EC solution compared to UW solution [20], the citrate/creatinine ratio was elevated in the UW group compared to the EC group during follow-up. These findings clearly demonstrated that retrieval conditions might influence renal medulla injury, and that UW solution is more efficient in reducing renal medullary damage than EC solution, even after prolonged CI [20,45,46]. Moreover, NMR spectroscopy was able to discriminate kidneys with significant renal damage more efficiently than conventional biochemical parameters and light microscopy [46].

Previously, ^1^H NMR-based metabolic profiling revealed mild and severe IRI in rat kidney grafts after 24 and 42 h of SCS, respectively [47]. Significantly decreased levels of polyunsaturated fatty acids and elevated levels of allantoin, a marker of oxidative stress, were found after 42 h of SCS. TMAO, a marker of renal medullary injury, and allantoin were significantly increased, correlating with the severity of histologic damage, while serum creatine (commonly used end point) values were not different between Tx groups [47]. In future clinical applications, quantitative metabolomics may help to distinguish between IRI, and early and chronic rejection.

In 2014, Bon et al. proposed a protocol for MP perfusate metabolomics analysis as a tool for the assessment of preserved kidney quality, to reduce the number of discarded organs and optimize patient management [49]. The potential of NMR to predict graft outcome by analyzing perfusates in a DCD pig model of kidney autoTx, over 22 h of HMP, was evaluated. Levels of several metabolites, including lactate, choline, or amino acids such as valine, glycine or glutamate, increased over time, whereas there was a reduction in total glutathione during this period. The changes in these biomarkers were less severe in grafts with better function recovery based on lower plasma creatinine levels determined after 3 months [49]. The authors concluded that the analysis of biomarkers during kidney HMP using NMR could be an interesting tool for assessing graft quality, and was compatible with clinical application.

Another study characterized the metabolic profile of porcine DCD kidneys using ^1^H NMR spectroscopy over 24 h of HMP, compared to traditional SCS controls [11]. The total amount of central metabolites, such as lactate, glutamate, fumarate, aspartate and acetate, observed in the HMP kidney system suggests a greater degree of *de novo* metabolic activity than during SCS [11]. Whilst the majority of glucose is metabolized into glycolytic endpoint metabolites, such as lactate, the presence of non-glycolytic pathway derivatives suggests that the metabolism during HMP is more complex than previously thought [50]. The maintenance of central metabolic pathways may contribute to the clinical benefits of HMP.

Supplemental oxygenation during HMP was proposed to restore cellular ATP levels and ensure metabolic activity in DCD kidney grafts [48]. More recently, using NMR combined with GC-MS, Patel et al. found that 18 h HMP of porcine DCD kidneys with high perfusate partial pressure of oxygen (PO_2_, 95%) results in a greater degree of aerobic metabolism at the end of MP, compared to active aeration (21%) [51]. Darius et al. [52] investigated the metabolic, functional, structural and flow dynamic effects of low and high perfusate PO_2_ (30% vs. 90%) during a continuous 22 h HMP in a porcine DCD kidney IRI autoTx model, confirming those findings. ^1^H NMR analysis was used to determine the concentration of metabolites within the circulating perfusate at the end of the perfusion. While this animal study did not yield any advantages for early graft function after high perfusate PO_2_, compared to low PO_2_, perfusate metabolic profile analysis suggested that high perfusate PO_2_ conditions supported the aerobic mechanism [52]. More effective MP strategies could reduce the harmful effects of IRI, hence improving outcomes of Tx.

Subsequently, NMR spectroscopy was used to examine the metabolic profile of the HMP perfusate, at 45 min and 4 h, from human cadaveric kidneys awaiting Tx [10]. In this study, promising discriminators between kidneys with DGF and those with immediate graft function (IGF) were identified. Glucose, inosine and gluconate concentrations were lower in DGF kidneys compared to IGF at both time points, while leucine concentrations were higher [10]. During kidney HMP, a significant portion of the metabolic activity persists—a currently poorly understood mechanism. Therefore, further research on the modification of harmful metabolic processes may improve graft-related outcomes, and consequently has the potential to modify ECD organs. Furthermore, it remains unclear how accurately levels of perfusate metabolites reflect intracellular activity.

The same research group later compared the metabolic profiles of human and porcine kidneys with regard to HMP-derived perfusate to determine whether the porcine model is a valid surrogate for human studies [53]. Out of 30 metabolites analyzed, 16 were present in comparable concentrations in the pig and human kidney perfusates. Only 3-hydroxybutyrate showed significantly different rates of concentration change [53]. It seems that pig and human kidneys during HMP appear to be metabolically similar, confirming the pig as a valuable model for further kidney-related studies.

### 3.4. Metabolomics in Liver Preservation

Rapidly increasing demands for liver Tx have caused severe shortages of donor liver organs [12]. Data on different preservation strategies suggest that MP is superior to SCS in improving short-term outcomes after human liver Tx, with a less clear effect in the longer term [12]. The use of metabolomics during liver preservation shows promising results in developing objective and reliable techniques to metabolically profile liver grafts and find reliable prognostic biomarkers prior to Tx (Table 4).

Hypothermic resuscitation perfusion of the preserved liver was capable of restoring high-energy nucleotides even after prolonged SCS time (48 h) [54]. The metabolic profiles of adenine nucleotides demonstrated a direct correlation between high ATP content prior to Tx and improved outcome in terms of liver function, as indicated by the normalization of serum enzyme levels and prothrombin time post Tx [61]. In porcine liver grafts, the levels of nucleotide triphosphates decreased to undetectable levels during 4 h of SCS, but regenerated after 2 h of oxygenated HMP, while glycolytic intermediates (3-phosphoglycerate and 2,3 diphosphoglycerate) increased significantly during SCS and subsequently declined following HMP [55]. Cellular damage, determined by the concentrations of glycerophosphorylcholine (GPC) and glycerophosphorylethanolamine (GPE), was minimal during SCS. However, upon HMP, the levels of GPC and GPE decreased, indicating a degree of cellular damage caused by reperfusion [55]. Interestingly, prolonged SCS (24 h) was associated with a significantly reduced (approximately 40%) liver capacity to regenerate ATP levels during hypothermic reperfusion when compared to the shorter SCS time (2 h) [56].

Gibelin et al. assessed liver graft function in isolated perfused rat livers after 24 h of preservation in EC vs. UW solution at 4 °C [57]. The transaminases levels were similar in these groups; however, the levels of lactate, pyruvate, succinate, citrate, aceto-acetate and b-hydroxybutyrate detected by proton NMR were significantly higher in the UW than in the EC group [57]. The analysis of metabolic profiles allows an efficient evaluation of liver graft preservation quality and the functional recovery during reperfusion.

The first study applying ^1^H NMR analysis to bile produced during NMP was published by Habib et al. [58]. This study revealed several changes in biliary constituents, between bile produced during retrieval and perfusion, as follows: (i) the concentration of bile acids, lactate, glucose and phosphatidylcholine increased, while (ii) the concentration of acetate decreased. These changes were more pronounced in DCD rabbit liver grafts compared to DBD grafts, although this did not reach statistical significance [58]. These metabolites may be potential markers of the extent of WI injury and the functional activity of machine-perfused liver grafts.

Previously, Fontes et al. described a new preservation modality for the liver, combining subnormothermic MP (SNMP; ∼21 °C) with hemoglobin-based oxygen carrier (HBOC) solution in a porcine orthotopic Tx model [60], analyzing over 600 tissue, perfusate and bile metabolites by GC-MS. The results revealed sustained metabolic activity (gluconeogenesis, albumin secretion, branched-chain amino acid secretion, urea production and ROS scavenging) during MP. Bile analysis over a 5-day period suggested that hydrophilic bile was secreted in the SNMP group, in contrast to hydrophobic bile documented in the SCS group. MP at 21 °C with the HBOC solution significantly improved liver preservation compared to SCS [60].

Later, Liu et al. proposed that the alanine and histidine measured by ^1^H NMR in HMP perfusate estimated WI injury in porcine liver grafts, and might be potential biomarkers of liver viability [59].

More recently, the metabolomic profiles, obtained by NMR, of back-table biopsies were significantly different in liver grafts with EAD [67]. The best discriminative metabolites, lactate and phosphocholine, were significantly associated with graft dysfunction, with excellent accuracy. The authors proposed the possibility of assessing the efficiency of graft resuscitation on MP by using these two markers in future studies [67]. Identifying metabolic biomarkers may enable the use of older donors and donors with longer ischemic times.

The first use of NMR spectroscopy on human liver samples was reported in 2005, determining metabolic profiles before organ retrieval, during HMP and after Tx [62]. The revealed variations in donor livers were consistent in most donors. First, GPC decreased in the majority of livers, suggesting increased cell turnover. Interestingly, in the graft that developed PGD, GPC remained stable, probably reflecting a lower degree of cellular activity, and therefore this substance might be a new biomarker for liver function [62].

Bruinsma et al. demonstrated the significant potential of MP combined with metabolomics as a clinical instrument for the assessment of preserved livers [63]. They applied SNMP (21 °C) on discarded human livers and determined changes by means of metabolic profiling with GC-MS and LC-MS, observing improvements in energetic cofactors and redox shifts, as well as the reversal of ischemia-induced alterations in specific pathways, including lactate metabolism and Krebs cycle intermediates. By this metabolomics approach, livers with similar metabolic patterns clustered based on the degree of injury [63]. This could help to identify organs that are suitable for Tx and those that should be discarded.

Karimian et al. compared the metabolomics of discarded steatotic human livers during 3 h of SNMP and NMP [64]. They found that steatotic livers replenish ATP storages more efficiently during SNMP than NMP. However, there is a significant depletion of glutathione during SNMP, likely due to the inability to overcome the high energy threshold needed for glutathione synthesis, highlighting the increased levels of oxidative stress in steatotic livers [64]. This study demonstrated that SNMP and NMP produce significantly different metabolomic profiles in liver grafts. More knowledge is needed to maximize the potential of both organ resuscitation techniques.

Raigani et al. recently analyzed the use of NMP in combination with metabolic profiling to elucidate the deficiencies in metabolic pathways in steatotic livers [65]. During NMP, energy cofactors increased in steatotic livers to a similar extent as in normal livers, but a significant lack in anti-oxidant capacity, efficient energy utilization and lipid metabolism was observed. Steatotic livers appeared to oxidize fatty acids at a higher rate, but favored ketone body production rather than energy regeneration via the Krebs cycle, leading to a slower lactate clearance and therefore higher transaminase levels in steatotic livers [65]. Currently, the lack of standard criteria for determining the graft suitability for Tx after MP remains a significant limiting factor as regards the clinical use of discarded human livers.

In 2020, Xu et al. proposed a small panel of metabolites involved in the purine pathway as promising biomarkers for the determination of human liver tissue quality before liver Tx [66]. Higher ratios of adenosine monophosphate/urate, adenine/urate, hypoxanthine/urate and alanine aminotransferase were associated with inferior graft quality (DBD vs. DCD) and outcomes (early graft function vs. EAD) post Tx. Moreover, a superior prediction ability as compared to a combination of conventional liver function and risk markers is proposed [66].

## 4. Conclusions

Innovative techniques of metabolic profiling, including NMR, GS-MS and LC-MS, have identified key pathways and mechanisms involved in organ damage during WI and CI, as well as IRI occurring during solid organ preservation. A growing number of experimental studies claimed metabolomics to be a promising tool for the assessment of graft quality as an original and reliable method, and which may easily be implemented in daily hospital routine. Although large-scale trials are needed, MP combined with metabolomics appears to be a potent tool for characterizing potential biomarkers to estimate graft-related outcomes prior to Tx. Moreover, biomarkers found in the perfusate, bile or urine are advantageous over organ biopsies, for being non-invasive and thus enabling more frequent and objective sampling. The currently available evidence on metabolic profiling during graft preservation suggests improved graft quality maintenance by HMP compared to traditional SCS, since significant metabolic activity is absent during SCS but not during HMP. Therefore, the regeneration of important metabolites, such as high-energy phosphate nucleotides, following a period of hypothermic perfusion in large, clinically related animal models has been proven to be feasible. HMP is able to support organ metabolism, and seems promising especially for long-term preservation. Other MP techniques (NMP and SNMP) revealed promising results too; however, further studies are necessary since the debate over the role of optimal preservation temperature continues. Moreover, more studies should focus on metabolic changes over time. In the future, we should progress toward organ-tailored preservation, whereby high-risk grafts can undergo assessment by metabolic profiling and re-conditioning prior to Tx; therefore, the maintenance of metabolic activity and organ function during preservation is an important factor. There is still a need for universal analytical techniques that are able to accurately—with appropriate sensitivity and specificity—identify and quantify the complete scope of metabolites in biological samples, thus enabling implementation in routine clinical practice.

## Figures and Tables

**Table 1:** Metabolomics in heart preservation for Tx.

Authors	Model	Metabolomics	Results
**Animal studies**			
Peltz et al., 2005 [28]	SCS vs. HMP with UW vs. Celsior solution (200 min) followed by 2 h reperfusion; Rats DBD without Tx; N = 10/group	^1^H and ^13^C NMR spectroscopy; Metabolites from tissue	*200 min of SCS* vs. *HMP* *HMP:* ↑ ATP, ~ADP, ↓ AMP, ~total adenylates; *HMP with UW* vs. *Celsior* *Celsior:* ↑ ATP, ↑ ADP, ↑ AMP, ↑ total adenylates; *SCS in UW* vs. *Celsior:* ~lactate, ~alanine
Rosenbaum et al., 2007 [29]	SCS vs. HMP (4 h) followed by 6 h reperfusion; Canine DBD with Tx; N = 6/group	^1^H and ^13^C NMR spectroscopy; Metabolites from perfusate and tissue	*4 h of SCS* vs. *HMP* *HMP:* ~glutamate enrichment, ↓ lactate, ↓ lactate/alanine ratio
Rosenbaum et al., 2008 [30]	SCS vs. HMP (4 h) followed by 6 h reperfusion; Porcine DBD with Tx; N = 4/group	^1^H and ^13^C NMR spectroscopy; Metabolites from perfusate and tissue	*4 h of SCS* vs. *HMP* *HMP:* ~glutamate enrichment, ↓ lactate, ↓ lactate/alanine ratio
Cobert et al., 2010 [31]	HMP with UW vs. Celsior^®^ solution (10 h); Canine DBD without Tx; N = 8	^1^H NMR spectroscopy; Metabolites from perfusate and tissue	*10 h of HMP with UW* vs. *Celsior*^®^*:* ~lactate, ~lactate/alanine ratio
Cobert et al., 2011 [32]	Retrograde HMP with different flow rates (20 min); Canine DBD without Tx; N = 6	^1^H NMR spectroscopy; Metabolites from tissue	*Lactate/alanine ratio* *Left atrium:* > 3 *Right ventricle:* >2 *Left ventricle:* ~1
Cobert et al., 2012 [33]	HMP with UW ± ^13^C labeled glucose vs. pyruvate (6 h); Rats DBD without Tx; N = 4/group	^1^H and ^13^C NMR spectroscopy; Metabolites from tissue	*HMP with UW (*glucose vs. pyruvate) *Glucose:* ↑ lactate/alanine ratio, ↓ lactate enrichment, ↓ alanine enrichment, ↓ glutamate enrichment
**Human studies**			
Cobert et al., 2014 [34]	SCS vs. antegrade HMP vs. retrograde HMP (12 h); DBD without Tx; N = 7–10/group	^1^H and ^31^P NMR spectroscopy; Metabolites from tissue	*SCS* vs. *HMP* *HMP:* ↓ lactate/alanine ratio, ↑ phosphocreatine/inorganic phosphate ratio, ↑ γ-ATP/inorganic phosphate ratio, ↑ phosphocreatine/γ-ATP ratio; *Antegrade* vs. *retrograde HMP:* ~lactate/alanine ratio, ~phosphocreatine/inorganic phosphate ratio, ~γ-ATP/inorganic phosphate ratio, ~phosphocreatine/γ-ATP ratio
Martin et al., 2019 [35]	WI and CI (6–480 min); Mice DBD with Tx, porcine and human DBD without Tx; N = >3/group	LC-MS; Over 100 metabolites from tissue	*30 min of WI* vs. *CI* *WI:* ↑ succinate, ↑ guanine, ↑ uracil, ↑ cystidine, ↑ guanosine, ↑ proprionylcarnitine, ↑ butyryl carnitine, ↑ hypoxanthine, ↑ lactate, ↓ aconitate, ↓ ATP/ADP ratio, ↓ glycogen; *0→60 min of WI* vs. *CI* *WI:* ↑ succinate, ↑ succinate/fumarate ratio; *CI:* ~succinate, ~succinate/fumarate ratio

ADP, adenosine diphosphate; AMP, adenosine monophosphate; ATP, adenosine triphosphate; CI, cold ischemia; DBD, donation after brain death; HMP, hypothermic machine perfusion; LC-MS, liquid chromatography–mass spectrometry; NMR, nuclear magnetic resonance; SCS, static cold storage; Tx, transplantation; UW, University of Wisconsin solution; WI, warm ischemia.

**Table 2:** Metabolomics in lung preservation for Tx.

Authors	Model	Metabolomics	Results
**Animal studies**			
Pillai et al., 1986 [38]	NMP with oxygenated blood (4 h); Porcine DBD without Tx; N = n.d.	^31^P NMR spectroscopy, Metabolites from tissue	*During NMP:* ~ATP, ~phosphodiester, ~inorganic phosphate, ~phosphomonoester; *During anoxia or ischemia:* ↓ ATP, ↑ inorganic phosphate
Jayle et al., 2003 [39]	SCS in UW vs. EC vs. PEG (6 h) followed by 75 min NMP; Porcine DBD without Tx; N = 5/group	^1^H NMR spectroscopy; Metabolites from bronchoalveolar lavage	*During NMP*: ↑ lactate, ↑ pyruvate, ↑ citrate, ↑ acetate; *SCS in UW* vs. *EC* vs. *PEG* *PEG:* ~ lactate, ~citrate, ↓ acetate, ↓ pyruvate
Peltz et al., 2005 [40,41]	SCS in Perfadex^®^ with ^13^C labeled glucose vs. pyruvate (6 and 24 h); Rats DBD without Tx; N = 4–6/group	^13^C NMR spectroscopy; Metabolites from tissue	*SCS in Perfadex*^®^*with glucose* vs. *pyruvate* *Pyruvate:* ↑ glutamate enrichment
Benahmed et al., 2012 [42]	HMP vs. SCS (3, 6 and 8 h); Porcine DCD without Tx; N = 8	^1^H NMR spectroscopy; 35 metabolites from tissue	*SCS:* ↑ majority of metabolites, ↓ glutathione, ↑ uracil; *HMP:* ~majority of metabolites, ~glutathione, ↓ uracil
**Human studies**			
Hsin et al., 2018 [43]	NMP (1 h and 4 h); Marginal grafts with Tx; N = 50	Mass spectrometry; 275 metabolites from perfusate	*PGD after 1 h of NMP:* ↑ palmitoyl-sphingomyelin, ↑ 5-aminovalerate, ↑ decanoylcarnitine; *PGD after 4 h of NMP:* ↑ N2-methylguanosine, ↑ 5-aminovalerate, ↑ oleamide, ↑ decanoylcarnitine

DCD, donation after circulatory death; EC, Euro-Collins solution; HMP, hypothermic machine perfusion; NMP, normothermic machine perfusion; NMR, nuclear magnetic resonance; PGD, primary graft dysfunction; PEG, polyethylene glycol solution; SCS, static cold storage; Tx, transplantation.

**Table 3:** Metabolomics in kidney preservation for Tx.

Authors	Model	Metabolomics	Results
**Animal studies**			
Richer et al., 2000 [45]	SCS in UW vs. EC (48 h); Porcine DBD with autoTx; N = 7/group	^1^H NMR spectroscopy; Metabolites from plasma and urine	*SCS in UW* vs. *EC after reperfusion* *UW in urine:* ↓ trimethylamidoxide/creatinine ratio, ↓ dimethylamine/creatinine ratio; *UW in plasma:* ↓ trimethylamidoxide
Hauet et al., 2000 [20]	SCS in UW vs. EC (48 h); Porcine DBD with autoTx; N = 14/group	^1^H NMR spectroscopy; Metabolites from plasma and urine	*SCS in UW* vs. *EC after reperfusion* *UW:* ↓ dimethylamine/creatinine ratio, ↓ trimethylamidoxide/creatinine ratio, ↑ citrate/creatinine ratio, ↓ acetate/creatinine ratio, ↓ lactate/creatinine ratio
Hauet et al., 2000 [46]	SCS in UW vs. EC (24 h) followed by 90 min reperfusion; Porcine DBD, single vs. multiorgan donor without Tx; N = 10/group	^1^H NMR spectroscopy; Metabolites from urine	*SCS in UW* vs. *EC after reperfusion* *UW in urine:* ~N-acetyl-β-D-glucosaminidase, ↓ lactate/creatinine ratio, ↓ acetate/creatinine ratio, ↓ trimethylamineoxide/creatinine ratio; *Single* vs. *multiorgan donor* *Single organ in urine:* ↓ lactate/creatinine ratio, ↓ acetate/creatinine ratio, ↓ trimethylamineoxide/creatinine ratio, ↑ amino acid, ↑ citrate
Serkova et al., 2005 [47]	SCS (24 vs. 42 h) ±Tx; Rats DBD with Tx; N = >6–8/group	^1^H NMR spectroscopy; Over 50 metabolites from plasma and tissue	*24* vs. *42 h of SCS after reperfusion* *24 h in blood: ~*creatinine, ↓ allantoin, ↑ polyunsaturated fatty acids; *24 h in tissue:* ↓ allantoin, ↓ trimethylamidoxide
Buchs et al., 2011 [48]	HOPE vs. SCS (8 and 18 h); Porcine DCD vs. DBD without Tx; N = 7	^31^P NMR spectroscopy; Metabolites from n.d.	*8→18 h of HOPE (DBD):* ~phosphomonoester, ~γ-ATP, ~α-ATP, ~β-ATP; *8→18 h of HOPE (DCD):* ↑ phosphomonoester, ↓ β-ATP; *8 h of SCS→8 h of HOPE (DBD):* ↓ phosphomonoester, ↑ γ-ATP, ↑ α-ATP, ↑ β-ATP; ↑ NADH; *18 h of SCS→8 h of HOPE (DBD):* ↓ phosphomonoester, ↑ γ-ATP, ↑ α-ATP, ↓ NADH
Bon et al., 2014 [49]	HMP (22 h); Porcine DCD with autoTx; N = 10	NMR spectroscopy; Metabolites from perfusate	*Inferior outcomes:* ↑ lactate, ↑ choline, ↑ amino acids (valine, glycine and glutamate), ↓ glutathione
Nath et al., 2016 [50]	HMP (6 and 24 h); Porcine DBD without Tx; N = 6	^1^H and ^13^C NMR spectroscopy; Metabolites from perfusate and tissue	*6→24 h of HMP:* ↑ lactate, ↑ alanine, ~acetate
Nath et al., 2017 [11]	HMP vs. SCS (24 h); Porcine DCD without Tx; N = 20	^1^H NMR spectroscopy; 26 metabolites from storage fluid/perfusate and tissue	*HMP* vs. *SCS* *HMP:* ↑ glutamate, ↑ myoinositol, ↑ lactate, ↑ formate, ↑ acetate, ↑ inosine, ↑ aspartate, ↑ niacinamide, ↑ fumarate
Patel et al., 2019 [51]	HOPE vs. aerated HMP (18 h); Porcine DCD without Tx; N = 16	^13^C NMR spectroscopy and GC-MS; Metabolites from perfusate and tissue	*HOPE* vs. *aerated HMP* *HOPE:* ↓ lactate, ↑ glutamate, ↑ ATP, ↓ alanine, ~citrate, ↑ succinate, ~malate
Darius et al., 2020 [52]	HMP ± 30% vs. 90% oxygen (22 h); Porcine DCD with autoTx; N = 28	^1^H NMR spectroscopy; 16 metabolites from perfusate	*HMP ± 30%* vs. *90% oxygen* *HMP + 90% oxygen:* ↓ acetate, ~adenine, ↑ alanine, ↑ aspartate, ↓ formate, ↓ gluconate, ↓ glutamate, ↑ glutathione, ↓ glycine, ↓ hypoxanthine, ~isoleucine, ↓ lactate, ~leucine, ↓ mannitol, ↓ myo-inositol, ↓ succinate
**Human studies**			
Nath et al., 2014 [53]	HMP (45 min and 4 h); Human vs. porcine DBD without Tx; N = 22	^1^H NMR spectroscopy; 30 metabolites from perfusate	*Human* vs. *porcine grafts Human:* ~conc. of 53.3% metabolites, ~change rate of conc. of 96.7% metabolites, ↑ change rate of conc. for 3-hydroxybutyrate
Guy et al., 2015 [10]	HMP (45 min and 4 h); DBD and DCD with Tx; N = 26	^1^H NMR spectroscopy; 28 metabolites from perfusate	*DGF* vs. *IGF* *DGF:* ↓ glucose, ↓ inosine, ↑ leucine, ↓ gluconate; IGF: ↑ glucose, ↑ inosine, ↓ leucine, ↑ gluconate

ATP, adenosine triphosphate; DBD, donation after brain death; DCD, donation after circulatory death; DGF, delayed graft function; EC, Euro-Collins solution; GC-MS, gas chromatography–mass spectrometry; HMP, hypothermic machine perfusion; HOPE, hypothermic oxygenated machine perfusion; IGF, immediate graft function; NADH, nicotinamide adenine dinucleotide; NMR, nuclear magnetic resonance; SCS, static cold storage; Tx, transplantation.

**Table 4:** Metabolomics in liver preservation for Tx.

Authors	Model	Metabolomics	Results
**Animal studies**			
Busza et al., 1994 [54]	SCS (48 h) in plasma-like vs. modified UW followed by 1 h HMP; Rats DBD without Tx; N = 10	^31^P NMR spectroscopy; Metabolites from whole organ	*After 48 h of SCS:* ↓ ATP, ↓ ADP, ↑ inorganic phosphate; *Plasma-like* vs. *modified UW solution (after reperfusion)* *Modified UW:* ↑ β-ATP/NAD(H) ratio, ↓ inorganic phosphate
Changani et al., 1996 [55]	SCS (4 h) followed by 2 h oxygenated HMP; Porcine DBD without Tx; N = 5	^31^P NMR spectroscopy; Metabolites from tissue	*During 4 h SCS:* ↓ nucleotide triphosphates, ↑ 3-phosphoglycerate, ↑ 2,3 diphosphoglycerate, ~glycerophosphorylcholine, ~glycerophosphorylethanolamine; *After 2 h of oxygenated HMP:* ↑ nucleotide triphosphates, ↓ 3-phosphoglycerate, ↓ 2,3 diphosphoglycerate, ↓ glycerophosphorylcholine, ↓ glycerophosphorylethanolamine
Changani et al., 1997 [56]	SCS (2 vs. 24 h) followed by 2 h oxygenated HMP; Porcine DBD without Tx; N = 6	^31^P NMR spectroscopy; Metabolites from perfusate	*2* vs. *24 h of SCS followed by 20 min oxygenated HMP* *24 h:* ↓ β-ATP, ↓ α-ATP; ↓ β-ATP, ↓ phosphomonoesters, ~inorganic phosphate, ↓ phosphodiesters, ↓ methylene diphosphonic acid; *2* vs. *24 h of SCS during 2 h oxygenated HMP* *24 h:* ↓ ATP
Gibelin et al., 2000 [57]	SCS in EC vs. UW solution (24 h) followed by 90 min reperfusion; Rat DBD without Tx; N = 10	NMR spectroscopy; Metabolites from perfusate	*SCS in EC* vs. *UW* *UW:* ↑ lactates, ↑ pyruvate, ↑ succinate, ↑ citrate, ↑ aceto-acetate, ↑ β-hydroxybutyrate
Habib et al., 2004 [58]	SCS (40 min) followed by 6 h NMP; Rabbits DBD vs. DCD without Tx; N = 4/group	^1^H NMR spectroscopy; Metabolites from bile	*During 6 h NMP:* ↑ bile acids, ↑ lactate, ↑ glucose, ↑ phosphatidylcholine, ↓ acetate; *DBD* vs. *DCD* *DBD:* ↓ bile acids, ↓ lactate, ↓ glucose, ↓ phosphatidylcholine, ↑ acetate
Liu et al., 2009 [59]	HMP (4 h); Porcine DCD (WI 4 h) vs. DBD without Tx; N = 11	^1^H NMR spectroscopy; Metabolites from perfusate	*During HMP:* ↑ lactate, ↑ alanine, ↑ histidine; *DCD* vs. *DBD* *DCD:* ↑ alanine, ↑ histidine
Fontes et al., 2015 [60]	SNMP with HBOC vs. SCS (9 h); Porcine DBD with Tx; N = 12	GC-MS; Over 600 metabolites from perfusate and bile	*SNMP* vs. *SCS* *SNMP in perfusate:* ↑ ascorbate, ↑ threonate, ↓ AMP, ↓ hypoxanthine, ↑ glucose, ↑ BCAA (valine, isoleucine and leucine), ↓ 12-hydroxyeicosatetraenoic acid; *SNMP in bile:* ↓ taurocholate, ↓ glycochenodeoxycholate, ↓ taurine, ↓ hypotaurine, ↑ lathosterol, ↑ campesterol, ↓ α-hydroxybutyrate, ↓ glycocholenate sulfate
**Human studies**			
Lanir et al., 1988 [61]	SCS (1.9–6.8 h); DBD and DCD with Tx; N = 25	LC-MS; Metabolites from tissue	*Prior to Tx* *Success:* ↑ ATP, ↑ ADP, ~AMP, ↑ ATP/ADP ratio, *↑* energy change, ↑ total adenine nucleotide content, ↑ guanosine diphosphate, ↑ uridine triphosphate, ~hypoxanthine, ~xanthine, ~NAD +
Duarte et al., 2005 [62]	SCS (9–13 h) DBD with Tx; N = 6	^1^H NMR spectroscopy; Metabolites from tissue	*Before retrieval* vs. *after SCS* vs. *post Tx* *PGF:* ↑ glycerophosphocholine; *PGD:* ~glycerophosphocholine; *Steatotic* vs. *non-steatotic grafts* *Steatotic:* ↑ triglycerides, ↓ phospholipids, ↓ amino acids, ↓ glucose, ↓ nucleotide-related compounds, ↓ clearance of UW
Bruinsma et al., 2016 [63]	SNMP (3 h); DCD vs. ECD without Tx; N = 21	LC-MS and GC-MS; 159 metabolites from perfusate and tissue	*0* vs. *3 h of SNMP:* ↓ carbohydrates (glucose-6-phosphate, fructose-6-phosphate, glycerol, xylitol, ribulose-5-phosphate, N-acetylmannosamine, maltose, raffinose, glyceric acid), *↑* amino acids (valine, threonine, proline, leucine, isoleucine and glutamine), *↑* citric acid, *↑* α-ketoglutarate, ↓ lactic acid, *↑* 2-hydroxyglutaric acid, *↑* urea, ↓ uric acid, *↑* oxoproline, ↓ N-acetyl-D-hexosamine; *0→3 h of SNMP:* ↑ amino acids (valine, threonine, leucine and isoleucin), ↓ lactic acid; *DCD* vs. *ECD* *DCD:* ↑ ATP, ↓ oxidized glutathione/glutathione ratio, ↓ NADH/NAD ratio
Karimian et al., 2019 [64]	SNMP vs. NMP (3 h); DBD/DCD (steatosis >30%) without Tx; N = 14;	LC-MS; Over 1600 metabolites from tissue	*After 3 h of SNMP* vs. *NMP* *SNMP:* ↓ glutathione, ↓ cysteinylglycine, ↑ 5-oxoproline, ↑ glutamate, ↓ cysteine, ↑ glycine, ↓ γ-glutamylcysteine, ↑ citrate, ↓ α-ketoglutarate, ↓ succinate, ↑ fumarate, ↑ malate, ↓ maltopentaose, ↓ maltotriose, ↓ maltose
Raigani et al., 2020 [65]	NMP (3 h); DBD/DCD (non-steatotic vs. steatotic) without Tx; N = 8;	LC-MS; Over 1600 metabolites from tissue	*Non-steatotic* vs. *steatotic after 3 h of NMP* *Non-steatotic:* ↑ glutathione, ↓ oxidized glutathione, ↑ N-acetylcysteine, ~ATP/ADP ratio, ↑ carnitine, ↓ β-hydroxybutyrate, ~citrate, ↓ α-ketoglutarate, ↑ succinate, ↑ fumarate, ↑ malate, ↑ glucose-6-phosphate, ~dihydroxyacetone phosphate, ↑ 2-phosphoglycerate, ↑ phosphoenolpyruvate, ↑ ribulose-5-phosphate, ↑ ribose-5-phosphate, ↑ xylulose-5-phosphate, ↑ eicosapentaenoic acid, ↑ docosapentaenoic acid, ↑ docosahexaenoic acid, ↑ cholesterol, ~taurine, ~choline
Xu et al., 2020 [66]	SCS (210–840 min); DBD vs. DCD with Tx; N = 47	LC-MS; Metabolites from tissue	*DBD* vs. *DCD* *DBD:* ↑ AMP/urate ratio, ↑ adenosine/urate ratio, ↑ adenine/urate ratio, ↑ hypoxanthine/urate ratio; *EGF* vs. *EAD* *EGF:* ↑ AMP/urate ratio, ~adenosine/urate ratio, ↑ adenine/urate ratio, ~hypoxanthine/urate ratio

ADP, adenosine diphosphate; AMP, adenosine monophosphate; ATP, adenosine triphosphate; BCAA, branched-chain amino acid; DBD, donation after brain death; DCD, donation after circulatory death; EC, Euro-Collins solution; ECD, extended criteria donor; EAD, early allograft dysfunction; EGF, early graft function; GC-MS, gas chromatography–mass spectrometry; HBOC, hemoglobin-based oxygen carrier; HMP, hypothermic machine perfusion; LC-MS, liquid chromatography–mass spectrometry; NADH, nicotinamide adenine dinucleotide; NMP, normothermic machine perfusion; NMR, nuclear magnetic resonance; PGD, primary graft dysfunction; PGF, primary graft function; SCS, static cold storage; SNMP, subnormothermic machine perfusion; Tx, transplantation; UW, University of Wisconsin solution; WI, warm ischemia time.

## Supplementary Materials

Supplementary materials can be found at https://www.mdpi.com/1422-0067/21/18/6607/s1.

## Abbreviations

ATP	adenosine triphosphate
CI	cold ischemia
DBD	donation after brain death
DCD	donation after circulatory death
DGF	delayed graft function
DMA	dimethylamine
EAD	early allograft dysfunction
EC	Euro-Collins solution
ECD	extended criteria donor
GC-MS	gas chromatography–mass spectrometry
GPC	glycerophosphocholine
GPE	glycerophosphorylethanolamine
HMP	hypothermic machine perfusion
IGF	immediate graft function
IRI	ischemia–reperfusion injury
LC-MS	liquid chromatography-mass spectrometry
MP	machine perfusion
NMP	normothermic machine perfusion
NMR	nuclear magnetic resonance
PEG	polyethylene glycol
PO_2_	partial pressure of oxygen
ROS	reactive oxygen species
SCS	static cold storage
SERCA	sarcoplasmic reticulum calcium adenosine triphosphatase
SNMP	subnormothermic machine perfusion
TMAO	trimethylamine-N-oxide
Tx	transplantation
UW	University of Wisconsin solution
WI	warm ischemia
