# Supplementary material for: The Role of Metabolomics in Current Concepts of Organ Preservation
Source: Int J Mol Sci. 2020 Sep 10;21(18):6607. doi: 10.3390/ijms21186607 (PMC7555311; doi:10.3390/ijms21186607)
Supplement: Supplementary file 1 [file ijms-21-06607-s001.zip › Supplementary Materials/Supp_material_Search_strategy.docx]

Full database specific search strategies:

SEARCH STRATEGY, PUBMED:

("Organ Transplantation"[MeSH Terms] OR "Transplantation"[MeSH Terms] OR (("Organ"[All Fields] AND "transplant"[All Fields]) OR "Organ transplant"[All Fields]) OR (("Organ"[All Fields] AND "transplantation"[All Fields]) OR "Organ transplantation"[All Fields]) OR (("Liver"[All Fields] AND "transplantation"[All Fields]) OR "liver transplantation"[All Fields]) OR (("kidney"[All Fields] AND "transplantation"[All Fields]) OR "kidney transplantation"[All Fields]) OR (("intestine"[All Fields] AND "transplantation"[All Fields]) OR "intestine transplantation"[All Fields]) OR (("heart"[All Fields] AND "transplantation"[All Fields]) OR "heart transplantation"[All Fields]) OR (("lung"[All Fields] AND "transplantation"[All Fields]) OR "lung transplantation"[All Fields]) OR (("pancreas"[All Fields] AND "transplantation"[All Fields]) OR "pancreas transplantation"[All Fields]) OR "Organ Preservation"[MeSH Terms] OR "Perfusion"[MeSH Terms] OR "Preservation"[All Fields] OR "Perfusion"[All Fields] OR (("Perfusion"[All Fields] AND "machine"[All Fields]) OR "Machine perfusion"[All Fields]) OR (("Cold"[All Fields] AND "Storage"[All Fields]) OR "Cold Storage"[All Fields])) AND (("Metabolomics"[MeSH Terms] OR "Metabolomics"[All Fields] OR "Metabolic profiling"[All Fields] OR "Metabolic profile"[All Fields])

Language: English
Time span: All years

SEARCH STRATEGY, EMBASE:

("Organ Transplantation" OR "Transplantation" OR "Organ transplant" OR "liver transplantation" OR "intestine transplantation" OR "kidney transplantation" OR "heart transplantation" OR "lung transplantation" OR "pancreas transplantation" OR "Organ Preservation Solutions" OR "Perfusion" OR "Machine perfusion" OR "Cold Storage") AND ("Metabolomics" OR "Metabolic profiling" OR "Metabolic profile")

Language: English
Time span: All years
