# Supplementary material for: The Role of Metabolomics in Current Concepts of Organ Preservation
Source: Int J Mol Sci. 2020 Sep 10;21(18):6607. doi: 10.3390/ijms21186607 (PMC7555311; doi:10.3390/ijms21186607)
Supplement: Supplementary file 1 [file ijms-21-06607-s001.zip › Supplementary Materials/Supp_material_Flowchart.docx]

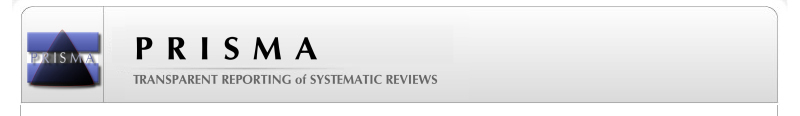
**PRISMA 2009 Flow Diagram**

`

Full-text articles excluded, with reasons
(n = 17)

Full-text articles assessed for eligibility
(n = 55)

Studies included in the review
(n = 38)

Records excluded
(n = 821)

Records screened
(n = 876)

Records after duplicates removed
(n = 876)

Additional records identified through other sources
(n = 0)

## Identification

## Eligibility

## Included

## Screening

Records identified through database searching
(n = 1387)
